# It Took Me a While to Figure out What Science I Really Wanted to Do

**DOI:** 10.1093/gbe/evac152

**Published:** 2022-11-04

**Authors:** T Martin Embley

**Affiliations:** Biosciences Institute, Newcastle University, England NE24HH, United Kingdom

We continue our biographical sketch series with one written by Martin Embley. Going forward, these biographical sketches will take two forms; some will be autobiographical and others will be in the form of an interview. If you have an interesting story to tell, then please contact the editors-in-chief Adam Eyre-Walker (a.c.eyre-walker@sussex.ac.uk) or Laura Katz (lkatz@smith.edu).

I was born in 1957 in Sunderland, a small coastal town in North-East England. Both of my parents left school at 14 and worked in local factories, my father maintained machinery, and my mother worked on a telephone assembly-line. My early school reports describe a child who was academically average and easily distracted, so it was a surprise when I passed the “11-plus” examination to attend the local Grammar school reserved for “academically able” children in 1960s England. Sadly, my annual school report cards over the next 7 years (my mother kept them) make disappointing reading. I liked, and did reasonably well in history and biology, but otherwise my marks were consistently average. Unlike many of my scientific friends, I can’t remember having a burning desire to be a scientist, but I do remember being fascinated by Jacob Bronowski’s book and TV series “The Ascent of Man.” In my final year at school, I failed to get the exam grades needed to go to a traditional university and ended up at Manchester Polytechnic studying for a degree in Biological Sciences. As far as I know, I was the first in my family to have any kind of higher education.

1970s Manchester was a large and exciting city, and the degree course was interesting and well taught. It included a research placement, so I ended up working in rural Kent for 6 months investigating the effects of fungicides on *Phytophora* infections of commercial soft fruits. I discovered that I enjoyed doing experiments and field work and began to think about research as a possible career. After my finals, I started a PhD project on bacterial fish diseases at Newcastle University. I spent the next 3 years traveling around the United Kingdom sampling the kidneys of hundreds of farmed trout and salmon for bacterial pathogens, while learning how to become an independent scientist. On completing my PhD, I was certain that I never wanted to dissect another fish. I’d become fascinated by evolution and the potential of molecular biology after reading about Carl Woese’s work using SSU rRNA sequences to reconstruct bacterial phylogenies. I wrote to some of the labs most active in the field, but I didn’t receive any positive replies. So, when I was offered a 1-year lectureship at North-East London Polytechnic (NELP), I jumped at the chance to gain some teaching experience while I continued to try and get a research position.

NELP was a former Further Education College, and still had a strong focus on applied science and training in vocational skills. I taught Industrial Microbiology to undergraduates and night classes in basic Microbiology to part-time students studying for technical qualifications. After a challenging first year, I was offered tenure; the United Kingdom was still in recession and jobs were scarce, so I accepted and ended up staying at NELP for the next 7 years. I was still hoping to do some research in evolution, but I didn’t have the skills or funding to set up my own molecular lab. To learn some molecular biology, I applied for a small “cultural exchange” grant in 1985 to visit Marian Mordarski, a close friend of my PhD supervisor Mike Goodfellow, in Wroclaw, Poland. Marian ran a lab using DNA techniques to classify bacteria and kindly offered to teach me his methods. Wroclaw still bore scars from the Second World War and whole streets would suddenly end in patches of wasteland because bombed houses had never been rebuilt. Food rationing was still in place, and I used to visit the “Pewex” hard currency shops to buy tins of American ham and German beer to supplement my rations.

**Fig 1:**
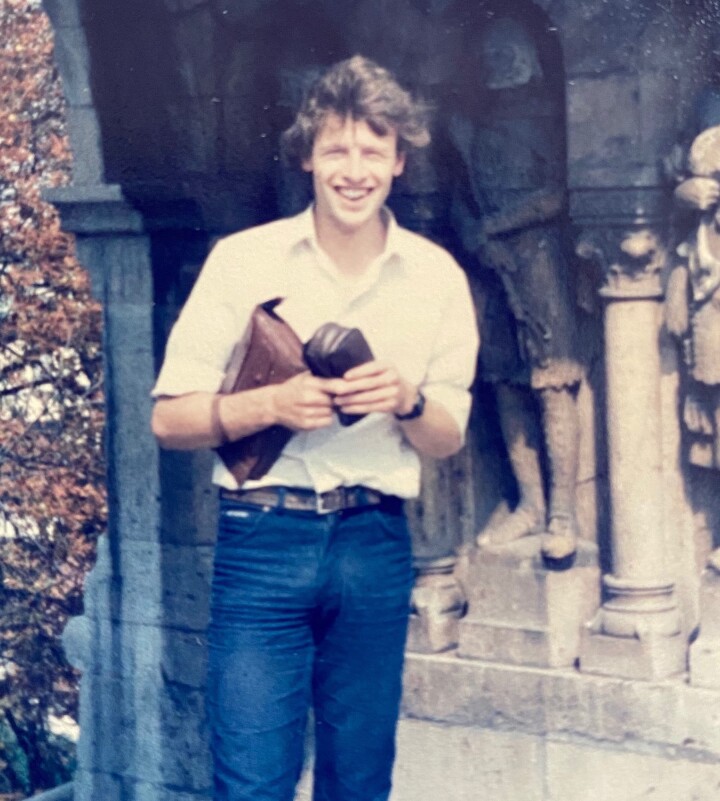
Martin in 1985 at conference in Hungary, just after he’d met Erko Stackebrandt, who had given him a lift to the conference.

My big scientific break came when Erko Stackebrandt visited Wroclaw on his way to a conference in Hungary. Erko had trained with Carl Woese and had already established the new SSU rRNA sequencing techniques in his lab in Kiel. I was going to the same conference as Erko, so he gave me a lift in his car and together we made a fascinating journey through 1980s Poland, Czechoslovakia, and Hungary (*Figure 1*). During the trip, I persuaded him to let me visit his lab in Germany to learn the new techniques. Collaborating with Erko was a revelation; he worked incredibly hard in a very focused and productive way. I stayed in his lab in Kiel for about 6 weeks during late 1986, and together with his talented PhD student Jan Smida, we generated enough data and ideas for two original papers (Embley et al. 1988a, 1988b).

Upon my return to London, I set up a molecular lab at NELP investigating the evolution of actinomycete bacteria funded by pharmaceutical companies who were interested in discovering new antibiotics. I also began to collaborate with microbial ecologists who wanted to use the new DNA methods to characterize natural communities without the need to isolate and culture the organisms. Bland Finlay introduced me to the fascinating world of anaerobic ciliates with hydrogen-producing mitochondria that host endosymbiotic methanogenic Archaea (Embley et al. 1992, 1995; Embley and Finlay 1994). My collaboration with Jim Prosser focused on ammonia-oxidizing bacteria in soils and sediments (Stephen et al. 1996) and with Dave Nedwell on methanogens and sulfate-reducing bacteria in marine and freshwater sediments (Munson et al. 1997; Purdy et al. 2001). These collaborations and their outputs started to get me noticed in the community, and in 1991, I was offered a research post at the Natural History Museum (NHM) in London.

My new job involved helping to establish a DNA lab at the museum, while also building my own research group. The film Jurassic Park was just about to appear and there was tremendous excitement about extracting “ancient DNA” from the Museum’s enormous collections to solve long-standing questions about evolution and biogeography. The museum let me choose my own research program, so I decided to work on the early evolution of eukaryotic cells and mitochondria. In the early 1990s, two ideas were central to views of early eukaryotic evolution. One was that the “three domains tree of life” was an accurate description of the relationships between eukaryotes and prokaryotes (Woese et al. 1990). The other was that some anaerobic and/or parasitic microbial eukaryotes that branched at the base of eukaryotes in this tree were primitively without mitochondria because they split from other eukaryotes before the mitochondrial endosymbiosis (Cavalier-Smith 1987). I’ve spent most of the last 30 years testing these ideas, and, while it has often been difficult and frustrating, it has also been tremendously exciting and a lot of fun.

The museum was a wonderful place to work and full of scientists who were passionate about evolution and how best to make evolutionary trees, so I quickly learned a lot about data analysis. Leaders in the field were always visiting and I had a chance to discuss science with Walter Fitch, James Lake, and Stephen Jay Gould, among others. I met Ford Doolittle at a Microbiology Meeting at Warwick in 1996 and discovered that we were interested in some of the same questions. Ford subsequently invited me to join the Canadian Institute of Advanced Research program in Evolutionary Biology as a foreign member, and for the next 10 years, I attended every one of the annual discussion meetings. These meetings were incredibly stimulating with a great mix of people active in the field of molecular evolution; they were also a prolific source of long-term friendships and collaborations.

Like most scientists running a research group, I’ve depended on attracting talented young people to do most of the actual work. Robert Hirt walked into the NHM and said he wanted to do a project mixing evolution and ecology. Robert was a hard-core molecular cell biologist who had worked on the exquisite molecular detail of membrane transport, and now he wanted to do something different. Over the past 25 years, we have built a research group together focusing on eukaryotic molecular and cell evolution using anaerobic and intracellular parasites as our model systems (Horner et al. 1996; Hirt et al. 1997, 1999). These species have proved to be a good choice, not only they have medical interest, making work on them more fundable, but also some of them, like Microsporidia, have highly reduced genomes and cell organelles (Williams et al. 2002). This makes Microsporidia excellent model for identifying the truly essential features of eukaryotic cells that cannot be lost.

In 2004, I moved back to North-East England to become professor of Molecular Evolution at Newcastle University. I loved working at the NHM, but I was tired of commuting on overcrowded unreliable trains and spending so little time with my young family. I was lucky enough to persuade Robert and most of my lab to come as well, and we continued to investigate the evolution of eukaryotes and mitochondria. During our time at Newcastle, I’ve been privileged to host a series of talented PhD students and post-doctoral fellows, who have done some exceptional work in collaboration with colleagues elsewhere in the United Kingdom and Europe (Hrdý et al. 2004; Cox et al. 2008; Goldberg et al. 2008; Tsaousis et al. 2008; Heinz et al. 2012; Dean et al. 2018; Williams et al. 2020). Their papers, together with the outstanding work of many other labs, have helped formulate a new view of eukaryotic evolution that is very different to that held in the 1990s (Embley and Martin 2006; Williams et al. 2013). For example, all eukaryotes are now thought to contain (or have contained) a mitochondrial homologue that generally functions in Fe/S protein biogenesis but may not make ATP, and the host for the mitochondrial endosymbiont, and hence the eukaryotic nuclear lineage, is thought to have originated from within the Archaea. Accordingly, eukaryotes are now commonly viewed as the product of an interaction between (at least) those two prokaryotic partners, and hence are not a primary domain of life.

**Fig 2:**
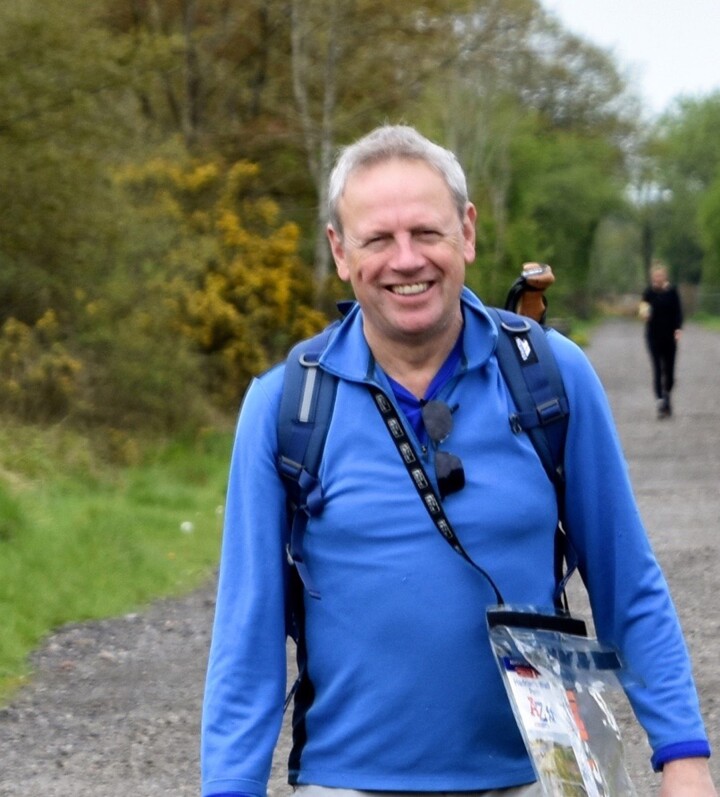
Martin recently walking the Hadrian’s wall path with his wife, Lynne.

I have always enjoyed a variety of interests outside of science. I love playing football and I still play every week with a group of fellow enthusiasts, some of whom I’ve known for over 40 years. I also watch live football even though my home-town team Sunderland is currently languishing in the lower divisions of British soccer. I’m still fascinated by history and archaeology and in retirement, I plan to travel with my wife to visit some of the countries and archaeological sites that I read about when I was at school. I’ve travelled all over the world as part of my job and it will be nice to revisit some of my favorite places together (*Figure 2*).

I’m sometimes asked what advice I’d give to someone starting out in science. Based upon my own experiences, I’d encourage anyone thinking about becoming a scientist to absolutely go for it, it can be a wonderful and rewarding career. It can be tough to stay in science, as it is very competitive and after a certain stage, depends as much upon an ability to secure regular funding as upon scientific talent. Nevertheless, many people do succeed in making it through these challenges, and one of the main tricks is not to give up. I’d also tell them not to worry if it takes a while to figure out what science you really want to do. In my own case, it wasn’t until I joined the museum that I identified some questions that really excited me. Lastly, always remember that doing any science properly takes a lot of time, care, and effort. So it’s worth spending time to identify a problem that is important and tractable, and where the solution will really merit all of the hard work needed to solve it.
